# The pan-PI3K inhibitor GDC-0941 activates canonical WNT signaling to confer resistance in TNBC cells: resistance reversal with WNT inhibitor

**DOI:** 10.18632/oncotarget.3568

**Published:** 2015-03-14

**Authors:** Huey-En Tzeng, Lixin Yang, Kemin Chen, Yafan Wang, Yun-Ru Liu, Shiow-Lin Pan, Shikha Gaur, Shuya Hu, Yun Yen

**Affiliations:** ^1^ Department of Molecular Pharmacology, Beckman Research Institute, City of Hope National Medical Center, Duarte, CA, USA; ^2^ Translational Research Core Laboratory, Beckman Research Institute, City of Hope National Medical Center, Duarte, CA, USA; ^3^ Joint Biobank, Office of Human Research, Taipei Medical University, Taipei, Taiwan; ^4^ Program for Cancer Biology and Drug Discovery, College of Medical Science and Technology, Taipei Medical University, Taipei, Taiwan; ^5^ Graduate Institute of Clinical Medicine, China Medical University, Taichung, Taiwan; ^6^ Division of Hematology/Oncology, Taichung Veterans General Hospital, Taichung, Taiwan; ^7^ School of Medicine, China Medical University, Taichung, Taiwan

**Keywords:** pan-PI3K inhibitor, triple-negative breast cancer, PI3K/AKT/mTOR pathway, WNT/beta-catenin pathway, drug resistance

## Abstract

The pan-PI3K inhibitors are one treatment option for triple-negative breast cancer (TNBC). However, this treatment is ineffective for unknown reasons. Here, we report that aberrant expression of wingless-type MMTV integration site family (WNT) and activated WNT signals, which crosstalk with the PI3K-AKT-mTOR signaling pathway through GSK3β, plays the most critical role in resistance to pan-PI3K inhibitors in TNBC cells. GDC-0941 is a pan-PI3K inhibitor that activates the WNT/beta-catenin pathway in TNBC cells through stimulation of WNT secretion. GDC-0941-triggered WNT/beta-catenin pathway activation was observed in MDA-MB-231 and HCC1937 cells, which are TNBC cell lines showing aberrant WNT/beta-catenin activation, and not in SKBR3 and MCF7 cells. This observation is further investigated in vivo. GDC-0941 exhibited minimal tumor inhibition in MDA-MB-231 cells, but it significantly suppressed tumor growth in HER-positive SK-BR3 cells. In vivo mechanism study revealed the activation of WNT/beta-catenin pathway by GDC-0941. A synergistic effect was observed when combined treatment with GDC-0941 and the WNT inhibitor LGK974 at low concentrations in MDA-MB-231 cells. These findings indicated that WNT pathway activation conferred resistance in TNBC cells treated with GDC-0941. This resistance may be further circumvented through combined treatment with pan-PI3K and WNT inhibitors. Future clinical trials of these two inhibitors are warranted.

## INTRODUCTION

Triple-negative breast cancer (TNBC) is a subtype of breast tumor that is negative for estrogen receptors (ER) and progesterone receptors (PR) and does not show over-expression of Her-2/neu (HER2). Higher occurrences of TNBC have been noted in younger and African-American women. Few drug choices with the exception of chemotherapy such as anthracycline, taxane and platinum are currently available for the treatment of TNBC. Patients with TNBC have a poor prognosis [1] because of the tendency for early relapse and visceral metastasis [2, 3]. TNBC is also insensitive to some of the effective therapies for breast cancer, including HER2-directed therapies, such as trastuzumab, and endocrine therapies, such as tamoxifen or aromatase inhibitors. Hence, treatment of TNBC remains a great challenge. Gene expression analysis has revealed six subtypes of TNBC [4], including two basal-like subtypes (BL1 and BL2) and immunomodulatory, mesenchymal (M), mesenchymal stem-like (MSL), and luminal androgen receptor (LAR) subtypes. Current and future treatment options include modulation of the DNA repair machinery, p53 family signaling, androgen receptor and MAPK/MEK signaling and PI3K/AKT/mTOR signaling. PI3K/AKT/mTOR inhibitors are one of these therapeutic regimens [5], and some of these inhibitors, such as GDC-0941, BKM120 and Everolimus, have been used as the therapeutic choice in recent clinical trials of TNBC (https://clinicaltrials.gov). It was recently reported that a PI3K inhibitor sensitized tumors to PARP inhibition by impairing BRCA1/2 expression [6].

Aberrant wingless-type MMTV integration site family (WNT) signaling has been noted in some human cancers, including colorectal cancer, ovarian cancer and breast cancer [7-9, 10]. The WNT/beta-catenin signaling pathway is important for the regulation of cell proliferation, migration, and differentiation. Therefore, this pathway is a significant regulator of embryonic development and tumorigenesis [11]. WNT proteins bind to Frizzled (FZD), which is a seven-pass transmembrane receptor protein, and to low-density lipoprotein receptor-related protein5/6 (LRP5/6) to activate the WNT/beta-catenin signaling pathway [12]. Activation of WNT/beta-catenin signaling is observed in TNBC and is associated with a poor clinical prognosis [9, 13-15]. Both the WNT/beta-catenin and PI3K pathways play critical roles in cancer cell biology. Moreover, the genes in one pathway interact directly with genes in the other pathway to integrate signaling in particular cell types. Integration of signaling from these two important pathways enhances cancerous growth in tumors and confers resistance against targeted therapy [16, 17]. This study reports the interaction between the PI3K/AKT/mTOR and WNT/beta-catenin signaling pathways in TNBC cells with pan-PI3K inhibitor treatment. This interaction conferred resistance to the pan-PI3K inhibitor GDC-0941 in the MDA-MB-231 and HCC1937 TNBC cell lines. Taken together, our findings elucidate a possible mechanism explaining the ineffectiveness of pan-PI3K inhibitors in TNBC treatment.

## RESULTS

### *In vitro* crosstalk between WNT and mTOR in MDA-MB-231 cells

**Figure 1:**
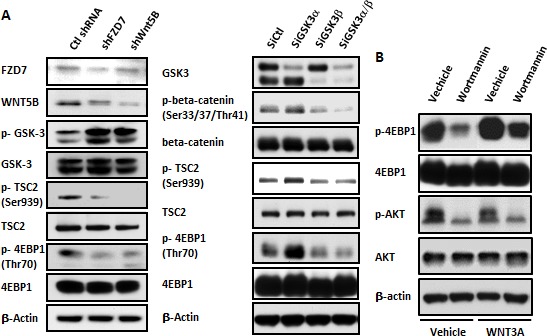
WNT/beta-catenin activity affected PI3K/AKT/mTOR signaling in MDA-MB-231 cells (A) The WNT/beta-catenin pathway was suppressed by infection with the shFZD7 and shWNT5B lentiviruses. A change in the PI3K/AKT/mTOR signaling pathway was detected in immunoblots 3 days after lentiviral infection (left). GSK3α or/and GSK3β was knocked down through the transfection of GSK3α or/and GSK3β siRNA at 30 nM. The phosphorylation of beta-catenin, TSC2 and 4EBP1 was examined via Western blotting 48 hr after transfection (right). (B) MDA-MB-231 cells were pretreated with or without WNT3A (75 ng/ml) for 24 hr. The PI3K inhibitor Wortmannin was added at a 1 μM concentration for 30 min. The cells were harvested for Western blot analysis.

Our previous paper identified FZD7 as the WNT/beta-catenin receptor and WNT5B as the WNT/beta-catenin ligand in MDA-MB-231 cells [11, 14]. We blocked FZD7 or WNT5B to evaluate changes in activity in PI3K/AKT/mTOR signaling and to investigate the crosstalk between the WNT/beta-catenin and PI3K/AKT/mTOR pathways in MDA-MB-231 cells. Lentiviruses targeting FZD7 and WNT5B (shFZD7 and shWNT5B, respectively) were used to suppress FZD7 and WNT5B. Western blot results indicated that WNT/beta-catenin signaling was attenuated, as demonstrated by enhanced GSK3β phosphorylation. However, downstream signaling of the PI3K pathway was also suppressed following the inhibition of WNT/beta-catenin signaling through shWNT5B or shFZD7 expression, as demonstrated by decreased phosphorylation of TSC2 and 4EBP1 (Figure 1A, left). GSK3β plays a role in bridging WNT/beta-catenin signaling with the mTOR pathway through interactions with TSC2 in certain type of cells [16]. Therefore, we examined the existence of this crosstalk function in the MDA-MB-231 TNBC cell line. We knocked down GSK3α/β using siRNA to address this question. GSK3β knock-down decreased beta-catenin phosphorylation and suppressed the phosphorylation of the PI3K pathway gene TSC2 and its response gene, 4EBP1 (Figure 1A, right). These results demonstrated that WNT signaling interfered with PI3K signaling in MDA-MB-231 cells, which implied that aberrant WNT signaling may compromise the effect of upstream PI3K inhibitors. Wortmannin is a potent PI3K inhibitor used in laboratory settings. We examined whether WNT could rescue the Wortmannin–induced suppression of AKT and mTOR. Figure 1B reveals that Wortmannin treatment decreased the mTOR signal, but the supply of WNT3A rescued 4EBP1 phosphorylation. The level of p-AKT did not change following WNT3A treatment, possibly because AKT is located upstream of TSC2. These results showed that excessive WNT may confer resistance to PI3K inhibitors, which may function through the cooperation between GSK3β and TSC2 in MDA-MB-231 cells.

### PI3K/AKT/mTOR inhibitors in MDA-MB-231, HCC1937, MCF-7 and SK-BR3 cells

**Figure 2:**
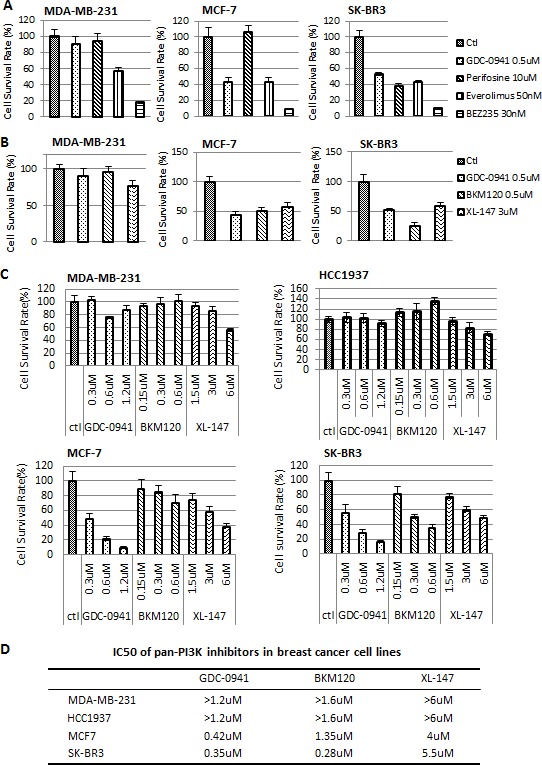
The efficacy of pan-PI3K, AKT and mTOR inhibitors in MDA-MB-231, MCF-7 and SK-BR3 cells (A) A total of 2,000 cells from each breast cancer cell line were seeded into each well of 96-well plates. The cells were treated with the indicated inhibitors and concentrations for 72 hr. Final surviving cells were enumerated using a DIMSCAN system. GDC-0941, pan-PI3K inhibitor; Perifosine, AKT inhibitor; Everolimus, mTOR inhibitor; and BEZ235, dual PI3K/mTOR inhibitor. The average cell survival rate ± SEM was plotted. (B) Using the same cellular density and incubation time, the survival cell rate was determined in MDA-MB-231 cells after treatment with the pan-PI3K inhibitors GDC-0941, BKM120 and XL-147 at the indicated concentrations. Data are represented as mean ± SEM. (C) Dose-treatment course of the pan-PI3K inhibitors in MDA-MB-231, HCC1937, MCF-7 and SK-BR3 cells. Cells were treated at the indicated concentrations for 72 hr and the survival cells were examined by DIMSCAN. Data were plotted as mean ± SEM. (D) IC50s of the pan-PI3K inhibitors in MDA-MB-231, HCC1937, MCF-7 and SK-BR3 cells. The cells were treated with GDC-0941 (0.1-1.2 μM), BKM120 (0.15-1.6 μM) and XL-147(1.5-8 μM) for 72 hr. Cell viability was assayed using DIMSCAN, and IC50s were calculated.

PI3K, AKT and mTOR inhibitors are used clinically for the treatment of breast cancer. We compared the efficiency of pan-PI3K, AKT and mTOR inhibitors in three breast cancer cell lines, MDA-MB-231, MCF7 and SK-BR3, which represent triple negative, ER-positive and HER2/Neu-positive breast cancers, respectively. GDC-0941 is a pan-PI3K inhibitor; Perifosine is an AKT inhibitor; Everolimus is an mTOR1 inhibitor; and BEZ235 is a PI3K/mTOR dual inhibitor. Proliferation assays revealed that SK-BR3 cells were sensitive to all of the PI3K, AKT and mTOR inhibitors tested in this study, but MCF7 and MDA-MB-231 cells were resistant to the AKT inhibitor Perifosine (Figure 2A). Only MDA-MB-231 cells were resistant to the pan-PI3K inhibitor (pan-PI3KI) GDC-0941. We tested GDC-0941, BKM120 and XL-147 in these three cell lines to investigate whether MDA-MB-231 cells were resistant to all of the available pan-PI3K inhibitors. All of these inhibitors failed to inhibit the MDA-MB-231 TNBC cell line. However, they all successfully suppressed the proliferation of MCF7 and SK-BR3 cells (Figure 2B). We added another triple negative breast cancer cell line, HCC1937 and treated these four cell line cells with different concentrations of pan-PI3K inhibitors to exclude the possibility that the resistance of MDA-MB-231 cells was not caused by an improper dose; and verify whether the resistance only occurs in MDA-MB-231 cells Figure 2C shows that all three pan-PI3K inhibitors suppressed the proliferation of MCF-7 and SK-BR3 cells in a dose-dependent manner, but there was no dose-dependent effect in MDA-MB-231 and HCC1937 cells. The IC50s for these inhibitors in MCF-7, SK-BR3, MDA-MB-231 and HCC1937 cells are listed in Figure 2D. These findings confirmed that the MDA-MB-231 and HCC1937 TNBC cell line cells are resistant to pan-PI3K inhibitors.

### An aberrant WNT signal induced resistance to pan-PI3K inhibitors in TBNC cell line cells

**Figure 3:**
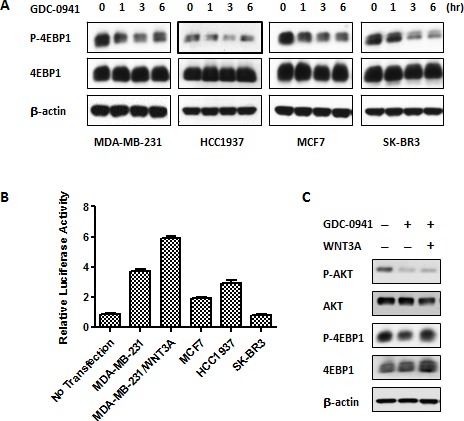
Aberrant activation of WNT/beta-catenin signaling conferred resistance in GDC-0941 in MDA-MB-231 cells (A) Cells were treated with 0.5 μM GDC-0941 for the indicated time course and then collected for Western blot analysis of p-4EBP1. (B) Cells were transfected with 400 ng of the TOPflash plasmid and 25 ng of the Renilla plasmid to perform dual luciferase assays in 24-well plates for various breast cancer cell lines. WNT3A (75 ng for 48 hr)-treated MDA-MB-231 cells served as a positive control; untransfected MDA-MB-231 cells were used as a negative control. Data are represented as mean ± SEM (n=3). (C) Cells were incubated with WNT3A at 75 ng/ml for one day prior to GDC-0941, and 0.5 μM GDC-0941 was added for 1 hr. The cells were then collected for Western blot analysis.

We demonstrated the activation of WNT/beta-catenin signaling and the interaction of this pathway with PI3K/AKT/mTOR signaling in MDA-MB-231 cells. Therefore, we sought to identify whether the resistance of MDA-MB-231 cells to PI3K inhibitors resulted from the crosstalk of the WNT and mTOR pathways. We performed immunoblotting to examine 4-EBP1 phosphorylation in MDA-MB-231, HCC1937, MCF-7 and SK-BR3 cells during the time course of GDC-0941 treatment. Notably, the suppression of p-4EBP1 reached a maximum level in 3 hr and started to rebound in 6 hr in MDA-MB-231 and HCC1937 cells, whereas p-4EBP1 remained inhibited at the last time point (6 hr) in MCF7 and SK-BR3 cells (Figure 3A). The TOPflash reporter system is widely used to monitor the activity of the TCF promoter, which is the downstream transcription factor in the WNT/beta-catenin pathway. We employed a TOPflash luciferase assay to examine WNT/beta-catenin signaling activity in MDA-MB-231, HCC1937, MCF7 and SK-BR3 cells. We found that MDA-MB-231 and HCC1937 cells exhibited higher WNT/beta-catenin activity than MCF-7 and SK-BR3 cells (Figure 3B). Robust WNT/beta-catenin activity was observed in MDA-MB-231 cells, and we speculated that the rebound of 4-EBP1 phosphorylation during extended GDC-0941 treatment might be due to an aberrant WNT/beta-catenin signal. We pre-treated cells with or without WNT3A for 24 hr and added GDC-0941 in the last hour to investigate this hypothesis. Treated cells were collected for Western blotting. We noted enhanced 4EBP1 phosphorylation with adding excessive WNT3A, which is a ligand of the WNT/beta-catenin pathway. These data confirmed that WNT3A rescued the attenuated mTOR activity induced by GDC-0941 treatment in MDA-MB-231 cells (Figure 3C). These data suggest that the resistance to PI3KIs observed in TNBCcells was caused by WNT/beta-catenin activation.

### GDC-0941 activates the canonical WNT signal in TNBC cells

**Figure 4:**
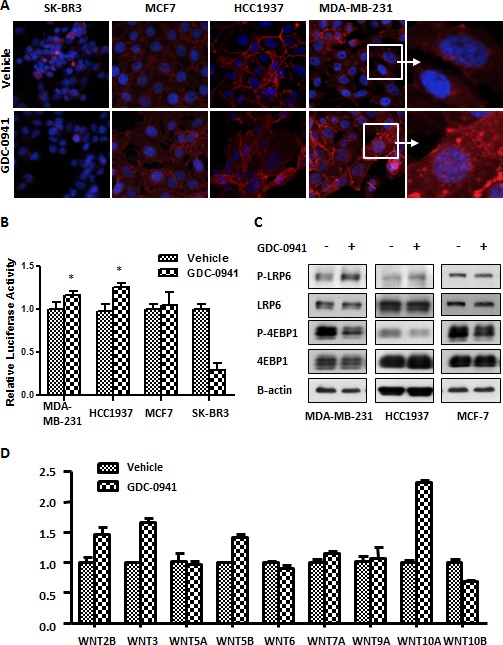
GDC-0941 activated Wnt signaling in MDA-MB-231 cells (A) Cells were seeded on coverslips the day before the experiment and treated with 0.5 μM GDC-0941 for 18 hr. The immunofluorescence (IF) of beta-catenin is indicated as red fluorescence, and double staining with DAPI is blue. Magnification: 400 X for the left three panels. The right panel shows enlarged images from the white squares. (B) Cells were transfected with 400 ng of the TOPflash plasmid and 25 ng of the Renilla plasmid for dual luciferase assays in 24-well plates in various breast cancer cell lines. Two days after transfection, the cells were treated with 0.5 μM GDC-0941 for 3 hr. The cells were then lysed for luminescence measurements. TOPflash activity was normalized against Renilla (n=3). * *p*<0.05. (C) MDA-MB-231, HCC1937 and MCF7 cells were treated with 0.5 μM GDC-0941 for 1 hr, and cell lysates were used for immunoblot analyses. (D) MDA-MB-231 cells were treated with 0.5 μM GDC-0941 for 24 hr. The relative mRNA expression of WNT ligands was analyzed using RT-qPCR. Black bars represent the SEM (n=3).

We further assessed the expression of WNT signaling in GDC-0941-treated breast cancer cells. The nuclear translocation of beta-catenin is the key step in WNT/beta-catenin pathway activation. Immunofluorescence (IF) staining revealed that higher level of beta-catenin was expressed in MDA-MB-231 and HCC1937 cells compared with SK-BR3 and MCF7 cells. Surprisingly, GDC-0941 treatment significantly enhanced the nuclear translocation of beta-catenin in MDA-MB-231 and HCC1937 cells compared with vehicle-treated cells (Figure 4A). We speculated that GDC-0941 activated the WNT/beta-catenin pathway in MDA-MB-231 and HCC1937 cells. Therefore, we applied a TOPflash luciferase assay to verify our initial observations of beta-catenin. TCF promoter activity increased in GDC-0941-treated MDA-MB-231 and HCC1937 cells, and this increase was significant (*p=0.017, p=0.013 respectively*). A similar increase was detected in MCF7 cells, but it was not statistically significant. However, GDC-0941 treatment greatly decreased WNT/beta-catenin signaling in GDC-0941-sensitive SK-BR3 cells (Figure 4B). WNT/beta-catenin pathway is triggered by the WNT ligand, which binds the FZD receptor to stimulate the phosphorylation of its co-receptor, LRP5/6. Western blotting showed that GDC-0941 stimulated LRP6 phosphorylation in MDA-MB-231 and HCC1937 cells, but not in MCF7 cells (Figure 4C), which implies that GDC-0941 initiated the WNT/beta-catenin signaling upstream in this pathway. Therefore, we evaluated WNT expression levels in GDC-0941- or vehicle-treated MDA-MB-231 cells. Classically, WNT1/2/3/3A/8A/8B/10A/10B are classified as canonical ligands, whereas WNT4/5A/6/7A/7B/11 are known as non-canonical ligands. The other WNTs, WNT2B/9A/9B/16, remain unclassified [18, 19]. We recently showed that WNT5B also stimulates the WNT/beta-catenin pathway and acts as a canonical WNT ligand in MDA-MB-231 cells [11]. RT-qPCR analysis of WNT expression in MDA-MB-231 cells demonstrated that WNT2B, WNT3, WNT5B and WNT10A were significantly up-regulated in GDC-0941 treated cells, and WNT10B was significantly down-regulated (Figure 4D). The strong elevation of three of four canonical ligands could explain the activation of WNT/beta-catenin signaling in MDA-MB-231 cells following GDC-0941 treatment. These findings demonstrated that GDC-0941 stimulated the transcription of WNTs, which promoted WNT-beta-catenin signaling in MDA-MB-231 cells.

### GDC-0941 exerted distinct effects in different tumor-derived cell line cells *in vivo*

**Figure 5:**
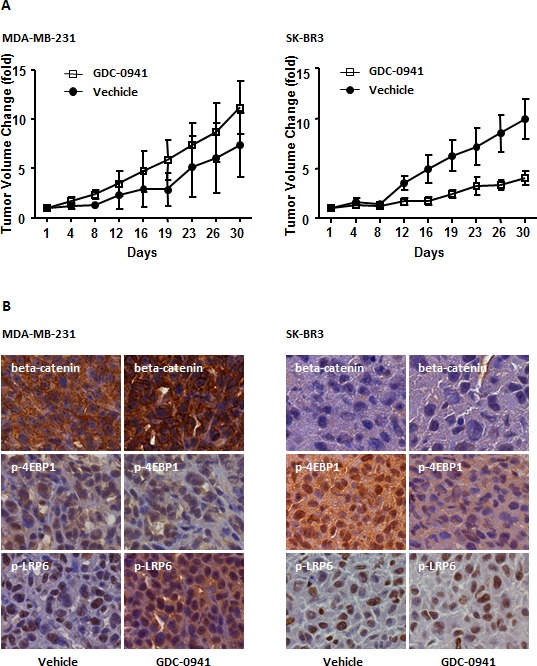
Effect of GDC-0941 on tumorigenesis *in vivo* (A) Subcutaneous tumors were generated by injecting 5 × 10^6^ cells of each cell line into the mammary pad of nude mice. The tumors were treated *in vitro* with 0.5 μM GDC-0941 for 24 hr. DMSO was used as a negative control. Tumor volume was measured every 4 days for 32 days. The results represent means ± SEM (n = 6). In an MDA-MB-231 breast cancer xenograft model, the GDC-0941-treated tumor volume was greater than that of vehicle-treated tumors, but statistical significance was not reached (*P* =0.2127) (left panel). In SK-BR3 xenograft tumors, GDC-0941 showed significant antitumor activity compared with vehicle-treated tumors (*P* = 0.0032). (B) The tumors were collected, and frozen tumor sections were used for immunohistochemistry staining of p-LRP6, p-4EBP1 and beta-catenin.

We found that aberrant WNT/beta-catenin signaling conferred resistance to the pan-PI3K inhibitor GDC-0941, and GDC-0941 amplified the WNT/beta-catenin signal in MDA-MB-231 cells. We next evaluated whether GDC-0941 functioned in a similar manner *in vivo*. We treated MDA-MB-231, MCF7 and SK-BR3 cells with GDC-0941 for 24 hr and transplanted the cells into nude athymic mice. Xenograft tumors successfully formed in MDA-MB-231 and SK-BR3 cells, but not in MCF7 cells. The failure of tumor formation in MCF7 cells may occur because these cells are incapable of developing tumors *in vivo*. Tumor volumes were measured every 4 days for 32 days. The average tumor volume generated from GDC-0941-treated MDA-MB-231 cells was greater than for vehicle-treated cells, although statistical significance was not achieved (*P=0.217*) (Figure 5A, left panel). However, GDC-0941 displayed strong antitumor activity in SK-BR3 xenograft tumors (*P=0.032*) (Figure 5A, right panel). These data confirmed that HER2-overexpressing tumors are sensitive to pan-PI3K inhibitors, whereas TNBC cell-derived tumors are resistant. We investigated the expression of p-LRP6, beta-catenin and p-4EBP1 in xenograft tumors to address the mechanism of resistance *in vivo*. Immunohistochemistry revealed enhanced LRP-6 phosphorylation and nuclear translocation of beta-catenin in GDC-0941-treated MDA-MB-231 tumors, but not in SK-BR3 tumors. The phosphorylation of 4-EBP1 was decreased in SK-BR3 tumors, but it was not altered in MDA-MB-231 tumors (Figure 5B). These data are consistent with our *in vitro* data, which suggest that GDC-0941 triggers a canonical WNT signal *in vivo* in MDA-MB-231-derived tumors.

### The combination of GDC-0941 and LGK974 showed a synergistic effect in MDA-MB-231 cells

**Figure 6:**
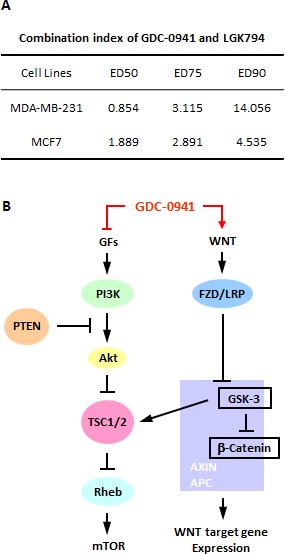
A WNT inhibitor enhanced the efficacy of GDC-0941 in MDA-MB-231 cells (A) MDA-MB-231 and MCF7 cells were treated with GDC-0941 combined with LGK974. The doses we used for GDC-0941 were 0.8 μM, 1.6 μM and 3.2 μM, and the doses for LGK974 were 0.75 μM, 1.5 μM and 3 μM, respectively. After incubation for 72 hr, proliferation assays were performed, and the synergistic effect was analyzed using CalcuSyn software (Biosoft). The combination index (CI) theorem defined the resulting calculation as an addictive effect, CI=1; synergism, CI<1 or antagonism, CI>1. (B) Schematic model for the potential pathways involved in the effects of GDC-0941 on MDA-MB-231 and HCC1937 cells.

PI3k inhibitors are one potential treatment for TNBC. However, we found that the pan-PI3K inhibitor GDC-0941 enhanced Wnt/beta-catenin signaling in the MDA-MB-231 TNBC cell line, which may be the reason for the drug resistance of these cells. Therefore, we combined GDC-0941 with a WNT antagonist, LGK974, which is currently being used in clinical trials for the treatment of breast cancer. LGK974 is an inhibitor of PORCN, which is required for the palmitoylation and subsequent secretion of WNT [20]. We tested LGK974 in MDA-MB-231 cells to examine its inhibition of the WNT/beta-catenin pathway. Only mild inhibition was detected at lower concentrations, but we chose LGK974 to perform the combination assay because of the limited availability of WNT inhibitors. The cell proliferation results showed that a synergistic effect was achieved at lower concentrations, which may be due to the weak efficacy of LGK974 in MDA-MB-231 cells. However, we did not observe any synergistic effect in MCF7 cells (Figure 6A). This finding raised the possibility that the addition of a WNT pathway inhibitor may reduce resistance to pan-PI3K inhibitors in TNBC therapeutics.

## DISCUSSION

The PI3K/AKT/mTOR signaling cascade plays a central role in cell biology, including proliferation, survival, metabolism, angiogenesis and genome stability, and this cascade is deregulated in diverse human cancers [21, 22]. Therefore, PI3K/AKT/mTOR pathway kinase inhibitors are widely investigated in laboratory and clinical trials. However, the therapeutic efficacy of a single agent is compromised clinically [23, 24]. The current study showed that TNBC cells were resistant to the pan-PI3K inhibitor GDC-0941 both *in vivo* and *in vitro*. The acquired resistance was induced by the crosstalk of GSK3β, a WNT/beta-catenin kinase, with TSC2, a PI3K/AKT/mTOR pathway gene, which modulated mTOR activity, as well as the stimulation of canonical WNT transcription, which further strengthened WNT/beta-catenin signaling. The overexpression of WNT/beta-catenin signaling overcame the inhibition of mTOR induced by the pan-PI3K inhibitor GDC-0941 (Figure 6B). Therefore GDC-0941 may actually potentiate cancer cell growth to cause drug resistance. Our findings provide good insight into the selection and combination of inhibitors for TNBC treatment. The outcomes of breast cancer have improved steadily, but TNBC remains the subtype with the worst prognosis [25, 26]. TNBC represents a highly heterogeneous class of breast cancer in terms of both the gene expression profile and the repertoire of genetic events. Therefore, the targeting of a subset, rather than a particular gene, will be critical for TNBC management [27]. We demonstrated that two cancer pathways are involved in TNBC, and both pathways need to be targeted simultaneously. However, there are numerous undetected signaling pathways that might be active and require suppression. This hypothesis can be exemplified by the potentiating of the antiproliferative activity of the PI3K inhibitor observed when other inhibitors, such as androgen receptor inhibitor, EGFR inhibitor and PARP inhibitors, are used together with PI3K inhibitors [28-30]. Therefore, a rational combination of inhibitors for suppressing certain sets of genes might be necessary for targeting therapies applied to treat TNBC.

The WNT signaling cascade is crucial for normal embryonic development and adult homeostasis in a variety of organ systems. Deregulation of WNT/beta-catenin signaling is related to many types of human cancer [31] and other diseases, such as metabolic disease, fibrosis and neurodegenerative disorders [32-34]. The activation of WNT/beta-catenin signaling is tightly modulated and may elicit unpredictable outcomes, depending on the context [35]. WNT pathway activation in human cancers is associated with gene mutations such as APC mutations [36, 37], AXIN mutations and beta-catenin mutations [38], epigenetic modifications such as methylation of the WNT negative regulator and secreted Frizzled-related proteins (SFRPs) [39] and positive or negative feedback control of gene expression in WNT/beta-catenin pathways. This study provides evidence that the pan-PI3K inhibitor GDC-0941 triggered the WNT/beta-catenin cascade in the MDA-MB-231 and HCC1937 TNBC cell line cells. This mechanism might be due to feedback regulation or GDC-0941-induced alterations in genome stability. However, elucidation of the detailed mechanism will require further studies.

## MATERIALS AND METHODS

### Cell culture and inhibitors

The breast cancer cell lines MDA-MB-231 (triple negative), HCC1937 (triple negative), MCF-7 (ER-positive) and SK-BR3 (HER2-positive) were purchased from American Type Culture Collection (ATCC) (Manassas, VA). MDA-MB-231 and MCF-7 cells were cultured in Dulbecco's Modified Eagle's Medium (DMEM), HCC1937 cells were cultured in RPMI140 and SK-BR3 cells were cultured in MaCoy's 5A with 10% fetal bovine serum and antibiotics. The cells were incubated at 37°C under 5% CO_2_. The compounds used in this study were purchased from the following vendors: Wortmannin (Sigma-Aldrich, St. Louis, MO), GDC-0941 (LC Laboratories, Woburn, MA), Perifosine (LC Laboratories, Woburn, MA), Everolimus (Sigma-Aldrich, St. Louis, MO), NVP-BEZ235 (LC Laboratories, Woburn, MA), BKM120 (Chemie Tek, Indianapolis, IN), XL147 (Chemie Tek, Indianapolis, IN), LGK974 (StemRd, Burlingame, CA), and WNT3A (R&D Systems, Minneapolis, MN).

### Cell growth and proliferation assays

Cells were seeded in 96-well plates in 100 μl of complete medium at a concentration of 2,000 cells per well the day before treatment. The cells were then treated with drugs for 72 hr for cytotoxicity assays. Viable cells were detected using 10 μg/ml fluorescein diacetate with 50 μl 0.5% eosin-Y/well to quench fluorescence in nonviable cells, followed by incubation at room temperature (RT) in the dark for 20 min. Fluorescence in viable cells was detected using DIMSCAN according to the manufacturer's instructions.

### siRNA transfection

Cells were suspended in 3 ml of medium and seeded into one well of a 6-well plate at a cell density of 2.5× 10^5^/ml the day before transfection. All small interfering RNAs (siRNAs) were purchased from Santa Cruz Biotechnology, Inc (Dallas TX). The stock concentration of each siRNA was 10 μM. Equal volumes of siRNA and the Lipofectamine RNAiMAX reagent (Life Technologies, Grand Island, NY) were diluted in Opti-MEM I medium without serum prior to mixing, according to the manufacturer's instructions, to form RNAi duplex–lipofectamine RNAiMAX complexes. These complexes were then incubated at RT for 10 min. The culture medium was removed during the incubation period and replaced with 2 ml of antibiotic-free complete medium. The above mixture was added drop-wise to each well after incubation, at a final siRNA concentration of 30 nM.

### Western blot analysis

Cell proteins were extracted from cells using RIPA buffer (Cell Signaling, Danvers, MA) containing a phosphatase inhibitor (Roche, Indianapolis, IN). Equal amounts of protein were loaded and separated through SDS-PAGE. The proteins were then transferred to a membrane, and the blot was blocked with 10% non-fat milk in TBST. The membranes were probed overnight at 4°C using the following antibodies: PI3 Kinase p110α, p-4E-BP1 (T70), 4E-BP1 (53H11), phosphor-AKT (Ser473), Akt (pan), p-GSK-3-alpha/beta (S21/9) (D17D2), GSK-3alpha/beta (D75D3), p-Tuberin/TSC2 (Ser939), p-beta-catenin (Ser33/34/Thr41), LRP6 (C5C7), p-LRP6 (S1490) (Cell Signaling, Danvers, MA), Myc (9E10) (Santa Cruz Biotechnology, Dallas, TX), FZD7 and β-actin (Sigma-Aldrich, St. Louis, MO). Appropriate antibodies were used for secondary antibody reactions. The ECL Plus Western Blot Detecting System (GE Healthcare, Piscataway, NJ) was employed to detect the presence of antibodies.

### Immunofluorescence assay

Cells were seeded on cover slips in six-well plates and allowed to adhere overnight until the cells reached 60%–70% confluence. The cells were subsequently treated with 0.5 μM GDC-0941 for 18 hr, followed by two washes with PBS buffer. Then, the cells were fixed using 4% paraformaldehyde for 25 min at room temperature, permeabilized with 1% Triton X-100 for 10 min, and incubated with a purified mouse anti-β-catenin antibody (BD Biosciences; 1: 500) at room temperature for 2 hr. The secondary antibody was an Alexa Fluor 680 goat anti-mouse IgG (Life Technologies). DAPI was used to visualize DNA. Fluorescence microscopy was performed in the City of Hope Microscope Core.

### Luciferase assay

MDA-MB-231, HCC1937, MCF-7 and SK-BR3 cells were distributed into 24-well plates the day before transfection. Cells at 80% confluence were co-transfected with a TCF-driven TOPflash reporter plasmid (Millipore) (400 ng) and control Renilla luciferase (25 ng) using 1.5 μl of Lipofectamine 2000 (Life Technologies). After 48 hr of co-culture, cells were lysed in 1X passive lysis buffer, and the supernatant was collected for dual luciferase activity measurements (Promega, Madison, WI). Firefly luciferase activity was normalized for each sample using Renilla luciferase activity as an internal control.

### Real-time RT-PCR

For complementary DNA (cDNA) synthesis, total RNA (1 mg) was transcribed using random hexamers (Life Technologies) and SuperScript III reverse transcriptase (Life Technologies) following the manufacturer's protocol. For DNA amplification, cDNA was denatured at 94°C for 30 s, and primers were annealed at 55–62°C for 30 s. DNA extension was performed at 72°C for 30 s for 24–30 cycles. Real-time RT–PCR analyses of WNT2B, WNT3, WNT5A, WNT5B, WNT6, WNT7A, WNT9A, WNT10A and WNT10B were performed using an iQ5 iCycler (Bio-Rad, Hercules, CA) according to the manufacturer's instructions. The amplification conditions consisted of an initial incubation at 95°C for 10 min, followed by 40 cycles of 95°C for 10 s and 59°C for 30 s. The data were analyzed using Bio-Rad iQ5 Optical System Software v2.0. All products yielded a single band of the predicted size.

### MDA-MB-231 and SK-BR3 cancer cell line xenografts

The female nude athymic mice (*National Laboratory Animal Center (NLAC), Taiwan*) used in these experiments were 8 weeks old on day 1 of the study. The animals received ad libitum water (reverse osmosis, 1 ppm Cl) and the PicoLab Rodent Diet 20 Modified and Irradiated Lab Diet®, which consisted of 20.0% crude protein, 9.9% crude fat, and 4.7% crude fiber. The mice were housed at the National Taiwan University Laboratory Animal Center (NTUMC) under a 12-hr light cycle at 21–23 °C and 60–85% humidity. Nude athymic mice were maintained in accordance with the procedures and guidelines of the Institutional Animal Care and Use Committee. Human MDA-MB-231 and SKBR3 breast cancer cells were maintained in DMEM or MaCoy's 5A medium containing 100 units/ml penicillin G sodium, 100 μg/ml streptomycin sulfate, 0.25 μg/ml amphotericin B, and 25 μg/ml gentamicin. The medium was supplemented with 10% fetal bovine serum and 2 mM glutamine. Cells were cultured in tissue culture flasks in a humidified incubator at 37 °C in an atmosphere of 5% CO_2_ and 95% air. There were two groups of 6 animals. One group was used as controls (DMSO treatment), and the other group received GDC-0941 treatment. Cells were treated *in vitro* with 0.5 μM/ml GDC-0941 for 24 hr before injection. For each animal, 5 million cells in 100 μl were injected under the mammary pad. Tumor size (in mm^3^) was calculated using the formula Tumor volume = (w^2^x l) / 2, where *w* = width and *l* = length in mm of the tumor. The tumor volume 24 hr after tumor implantation was taken as the baseline in each animal, and tumor size was measured every 4 days for 32 days.

### Statistical analysis

Significant differences were determined using the ANOVA test, and the results are expressed as means ± SEM (*n* ≥3). *P* < 0.05 reflected significant differences.
